# Effectiveness and Safety of Shorter Incontinence Slings

**DOI:** 10.1007/s00192-024-05971-5

**Published:** 2024-11-13

**Authors:** Kjersti Rimstad, Sissel Hegdahl Oversand, Marie Ellström Engh, Rune Svenningsen

**Affiliations:** 1 Department of Obstetrics and Gynaecology, Akershus University Hospital, PO box 1000, 1478 Lørenskog, Norway; 2 Department of Gynaecology, Oslo University Hospital, Oslo, Norway; 3 The Norwegian Female Incontinence Registry, Oslo University Hospital, Oslo, Norway; 4 Faculty of Medicine, University of Oslo, Oslo, Norway

**Keywords:** Follow-up studies, Persistent postsurgical pain, Registries, Suburethral slings, Transobturator suburethral tape, Stress urinary incontinence

## Abstract

**Introduction and Hypothesis:**

Traditional slings, tension-free vaginal tape obturator inside-out (TVT-O) and tension-free vaginal tape (TVT), have well-documented continence outcomes but can cause serious complications. This study was aimed at evaluating whether slings with less synthetic material, Ajust™ and TVT-O Abbrevo™ (TVT-A), have comparable 6– to 12-month failure and complication rates, including risk of prolonged postoperative pain, compared with traditional slings.

**Methods:**

A registry study from the Norwegian Female Incontinence Registry (NFIR) including 611 Ajust™, 2,772 TVT-A, and 18,612 traditional slings was carried out. Preoperative, surgical, and 6– to 12-month follow-up data from the period 2009–2021 were used. Objective failure was defined as ≥ 1-g leakage on standardized cough-jump stress test. Subjective failure was defined as stress index-score ≥ 3 on a validated questionnaire. Prolonged postoperative pain was defined as lasting > 3 months.

**Results:**

At first follow-up after 6–12 months, the groups differed significantly. Objective failure rates were as follows: Ajust™ 15.4%, TVT-A 13.5%, and traditional slings 7.3%, *p* < 0.01. Subjective failure rates were as follows: Ajust™ 23.4%, TVT-A 23.8%, and traditional slings 18.8%, *p* < 0.01. Shorter slings had fewer overall complications (Ajust™ 4.9% vs TVT-A 6.5% vs traditional slings 9.3%, *p* < 0.01), but did not have less prolonged postoperative pain (TVT-A: 1.4% vs Ajust™ 0.8% vs traditional slings 0.7%, *p* < 0.01 < 0.01). All presented outcomes remained significant after adjusting for differences at baseline.

**Conclusions:**

Shorter slings have inferior subjective and objective continence outcomes at 6–12 months, but fewer overall complications except for prolonged postoperative pain.

**Supplementary Information:**

The online version contains supplementary material available at 10.1007/s00192-024-05971-5

## Introduction

Stress urinary incontinence affects approximately 1 in 3 parous women and has a significant impact on quality of life (QOL) [1, 2]. Affected women often limit their physical activity out of fear of leaking, something that may also impair sexual function [3]. The traditional surgical approach for treating female stress urinary incontinence is to restore suburethral support by implanting a polypropylene mid-urethral synthetic sling. Traditional slings are placed under the urethra and passed either in front of the bladder through the pelvis to the lower abdomen (tension-free vaginal tape [TVT]) [4], or to the side of the pelvis in the groin area (tension-free vaginal tape obturator inside-out [TVT-O]) [5]. TVT and TVT-O have well documented short- and long-term continence outcomes and acceptable complication rates, also with regard to the risk of prolonged postoperative pain [1, 6, 7]. Most women treated with these traditional slings therefore obtain substantial improvement in QOL [1]. However, some women experience severe complications with both TVT and TVT-O, such as chronic pelvic pain, urinary retention, superficial and deep infections, erosions, and hematomas, whereas other complications are more specific to the area of sling placement, such as bladder perforations with TVT and groin pain with TVT-O [1, 6–9]. Focus in the media by patient organizations on the risk for prolonged pain due to the use of synthetic vaginal implants, as well as lawsuits against manufacturers, have made some countries revert to older, less effective surgical methods to avoid synthetic material altogether [10, 11].

With shorter slings, less synthetic material is used, with the goal of achieving similar continence outcomes to traditional slings, but with fewer severe complications [12, 13]. Several Norwegian hospitals shifted to shorter slings as early as 2009, initially using the single-incision mini-sling Ajust™ and from 2016 the intermediate sling TVT-O Abbrevo™ (TVT-A) [14, 15].

There are to date few published studies evaluating the effectiveness and complication rates of shorter slings, with adequate patient numbers for robust statistics [16–19]. Data on all female incontinence surgeries performed in Norway since 1998 are available through the national mandatory Norwegian Female Incontinence Registry (NFIR).

This study was aimed at using national registry data from the NFIR to compare failure rates at first follow-up 6–12 months after surgery for Ajust™, TVT-A, and traditional slings. Moreover, we wanted to compare complication rates, with a particular focus on prolonged postoperative pain.

## Materials and Methods

This was a registry study using anonymous data from the national NFIR, which is a mandatory quality registry for all female incontinence surgeries in Norway. All public hospitals and most private hospitals prospectively report their preoperative, surgical, and first follow-up data 6–12 months after surgery to the registry [20, 21]. The present study investigated data from women who underwent surgery with Ajust™, TVT-A, retropubic TVT, or TVT-O during the years 2009, when the first use of an Ajust™ was recorded in the registry, until 2021, with follow-up data through 2022. Preoperative data, surgical data, and data from first follow-up at 6–12 months were used. To avoid any possibility of indirect identification, the NFIR removed hospital names and instead categorized hospitals based on annual volume of surgical female incontinence procedures (low volume < 30, medium volume 31–99, or large volume ≥ 100). The date of surgery was given as year of surgery only. TVT and TVT-O have been used for the longest time in Norway and offer comparable short-term results and complication rates [1, 8]. For the majority of analyses these two procedures were grouped together as a reference group named “traditional slings” to emphasize potential differences between shorter and longer slings. However, as TVT and TVT-O differ somewhat regarding complication types, we also performed separate comparative analyses for this outcome.

Both surgical method and sling type are registered in NFIR. In addition, it is possible to add free text regarding the type of sling used. When there were discrepancies between these three variables, we cooperated with the NFIR administration and defined a hierarchy to decide on which variable was most likely correct. In the case of discrepancies, the sling type described in free text would trump the variable sling type, which again would trump the variable surgical type. The variable “sling type” was first added to the registry in 2015; thus, for entries between 2009 and 2015, free-text descriptions of sling type trumped surgical type if there was a discrepancy.

**Fig. 1:**
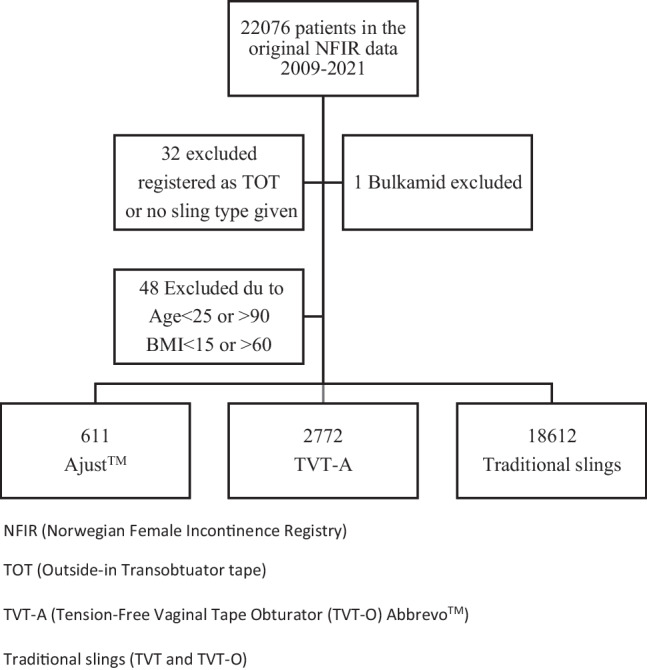
Flow chart of exclusion criteria FAST 1. (*NFIR* Norwegian Female Incontinence Registry, *TOT* outside-in transobturator tape, *TVT-O* tension-free vaginal tape obturator, *TVT-A* TVT-O Abbrevo™). TVT and TVT-O were grouped together as traditional slings

The original data set contained data from 22,076 patients with preoperative data, postoperative data, and data from the first follow-up performed 6–12 months after surgery. Eighty-one women had to be excluded as the sling type could not be determined. Furthermore, we decided to exclude women registered with age at surgery < 25 or > 90 years and women with body mass index (BMI) < 15 or > 60 kg/m^2^ as these, according to clinical practice, were likely erroneous entries (Fig. 1).

All hospitals reporting to the registry use a validated NFIR questionnaire filled out by the patients before surgery and at all consecutive follow-ups [22]. This questionnaire contains clusters of questions that generate indices describing the degree of stress and urgency urinary incontinence symptom bother and differentiates stress urinary incontinence symptoms from urgency urinary incontinence symptoms. The stress urinary incontinence index score ranges from 0 to 12, and the urgency urinary incontinence index score ranges from 0 to 8. Higher scores signify more symptoms. To access the complete questionnaire, see the Supplementary Material (Appendix 1).

Primary outcomes were short-term (6–12 months) subjective and objective failure rates. Subjective failure was defined as stress urinary index score ≥ 3, in accordance with the definition used by the NFIR in the national reports. Objective failure was defined as ≥ 1 g leakage on a standardized cough-jump pad-weighing stress test. This cough-jump pad-weighing stress test consists of three forceful coughs in the standing position followed by 20 jumping jacks with a 300-ml bladder volume [23]. The test has been validated and is used as an objective test for stress urinary incontinence before surgery and at all subsequent follow-ups by all hospitals reporting to the NFIR.

Secondary outcomes were total complication rates, including all types of sling-related complications defined by the NFIR. Prolonged postoperative pain was defined as pain lasting more than 3 months after surgery, given the options “no pain, or pain for less than 3 months,” “pain more than 3 months,” “pain more than 16 months,” and “pain more than 3 years.” The questionnaire contains no numerical scale quantifying postoperative pain. Treatment dissatisfaction rate, rates of de novo urgency, and persistent and worsened urgency incontinence were also compared.

To evaluate if prolonged pain was associated with BMI at the time of surgery, we compared women with BMI less than 20 kg/m^2^ or 25 kg/m^2^ with those with BMI ≥ 20 kg/m^2^ or ≥ 25 kg/m^2^ respectively. Treatment dissatisfaction was calculated as the percentage of women not answering “very satisfied” with treatment on a five-point Likert scale in the validated NFIR questionnaire using the categories: “very satisfied,” “moderately satisfied,” “neither satisfied nor dissatisfied,” “moderately dissatisfied,” or “very dissatisfied” [22]. De novo urgency incontinence was defined as urgency urinary incontinence index score ≥ 1 at the 6- to 12-month follow-up in women with a preoperative urgency urinary incontinence score of 0. Persistent urgency incontinence was defined as urgency urinary incontinence index score ≥ 1 both before and after surgery. Worsening of urgency incontinence was defined as any increase in urgency urinary incontinence index after surgery.

Data were only extracted from the NFIR for women who had signed a written consent form allowing their data to be used for research. The study was also approved by the NFIR Advisory board and the institutional Personal Data Officer.

Statistical analyses were performed using the Statistical Package for the Social Sciences (SPSS 29.0; IBM, Armonk, NY, USA). Differences between groups were analyzed using the Chi-squared test or Fisher’s exact test when appropriate for categorical variables and one-way analysis of variance for continuous variables. Logistic regression analyses were performed to adjust for group differences at baseline. We adjusted for age at surgery (years), body mass index at surgery stratified according to the World Health Organization (WHO) classification (< 24.9, 25.0–29.9, 30.0–34.9, ≥ 35.0), hospital annual surgical volume (< 30, 31–99, ≥ 100), preoperative urgency urinary index scores (0, 1–4, 5–8), preoperative stress urinary index scores (0–6 [< 33.3 percentile], 7–9 [33.3–66.7 percentile], 10–12 [≥ 66.7 percentile]), preoperative objective leakage on the cough-jump stress test (0–19 [< 33.3 percentile], 20–54 [33.3–66.7 percentile], > 54 [≥ 66.7 percentile]), and the presence of any complication. Missing values were handled by data removal for each variable. No imputation statistics were performed.

## Results

Preoperative, surgical, and 6- to 12-month follow-up data from 22,076 women who had had surgery with the four sling types during the period 2009–2021 with follow-up data through 2022 were extracted from the NFIR. The final dataset used for the analyses after excluding erroneous entries contained 611 single-incision minislings Ajust^TM^, 2,772 intermediate slings TVT-A, and 18,612 traditional slings (TVT-O/TVT; Fig. 1). There were small but significant differences between groups at baseline (Table 1). Mean age was lower, mean BMI higher and mean leakage larger at the time of surgery for shorter slings (*p* < 0.01; Table 1). Objective and subjective failure rates at 6–12 months were significantly higher for the two shorter slings. Objective failure was as follows: Ajust™ 15.4%, TVT-A 13.5%, and traditional slings 7.3% (*p* < 0.01). Subjective failure was as follows: Ajust™ 23.4%, TVT-A 23.8%, and traditional slings 18.8% (*p* < 0.01). Having adjusted for group differences at baseline, shorter slings still had a significantly higher risk of subjective and objective failure at 6–12 months than traditional slings (Table 2). The adjusted odds ratio (aOR) for subjective failure with Ajust™ and TVT-A were 1.49 (95% CI 1.17–1.91) and 1.45 (95% CI 1.28–1.65) respectively. For objective failure the aORs for Ajust™ and TVT-A were 2.86 (95% CI 2.03–4.03) and 1.42 (95% CI 1.18–1.71) respectively (Table 2). Women operated on with shorter slings had a significantly higher treatment dissatisfaction rate: Ajust™ 17.5%, TVT-A 18.3%, and traditional slings 14.3% (*p* < 0.01). Traditional slings had more registered complications: 9.3% vs 6.5% (TVT-A) and 4.9% (Ajust™; *p* < 0.01; Table 3). However, prolonged pain > 3 months after surgery occurred more often in the TVT-A group (1.4%; Table 3). Differences in prolonged postoperative pain remained significant after adjusting for group differences at baseline (Table 2), and were not dependent on BMI (Supplementary Table 1).

**Table 1:** Baseline characteristics of study participants at the time of surgery

Continuous variables, mean (SD)	Total, *N* = 21,995	TVT-A, *N* = 2,772	Ajust™, *N* = 611	Traditional slings^a^, *N* = 18 612	*p* value*
Age, years	50.7 (11.1)	49.8 (10.9)	50.0 (10.7)	50.9 (11.1)	* < 0.01*
Missing	0.2%	0.1%	0.5%	0.2%	
BMI, kg/m^2^	26.5 (4.5)	26.8 (4.6)	26.5 (4.4)	26.5 (4.5)	* < 0.01*
Missing	10%	18.7%	21.3%	8.3%	
Preoperative stress test, gram	48.5 (46.7)	53.3 (47.0)	74.9 (55.8)	46.9 (46.0)	* < 0.01*
Missing	5.4%	10.5%	2.1%	4.7%	
Preoperative Stress Urinary Incontinence Index Score (0—12)	8.3 (1.9)	8.4 (1.9)	8.5 (1.9)	8.3 (1.9)	* < 0.01*
Missing	7.9%	8.5%	11.3%	7.7%	
Preoperative Urgency Urinary Incontinence Index Score (0–8)	3.1 (2.2)	3.1 (2.2)	3.2 (2.2)	3.1 (2.2)	0.23
Missing	3.0%	3.4%	4.3%	3.0%	
Preoperative postvoid residual volume, ml	10.8 (20.7)	10.2 (20.2)	9.8 (20.1)	11.0 (20.7)	0.077
Missing	0.3%	0.3%	0%	0.3%	
Preoperative maximum flowrate (Q_max_), ml/s	29.5 (9.7)	29.2 (9.5)	27.8 (8.7)	29.6 (9.8)	* < 0.01*
Missing	17.1%	24.6%	15.5%	16.0%	
24-h pad test, g	66.6 (103.1)	65.8 (97.1)	79.1 (126.3)	66.5 (103.4)	0.167
Missing	27.5%	37.6%	61.4%	24.9%	
Categorical variables, %					
Concomitant Pelvic Organ Prolapse Surgery	1.3%	1.4%	2.1%	1.3%	0.203
Missing	1.0%	0.1%	0%	1.2%	
Preoperative MUCP < 20 cm H_2_O	4.4%	11.0%	1.8%	4.1%	* < 0.01*
Missing	79.6%	90.5%	55.6%	78.7%	

*BMI* body mass index, *SD* standard deviation, *MUCP* maximum urethral closure pressure, *TVT* tension-free vaginal tape, *TVT-O* tension-free vaginal tape obturator, *TVT-A* TVT-O Abbrevo™

*One-way analysis of variance or Chi-squared when appropriate

^a^Traditional slings (retropubic TVT and TVT-O)

Significant *p*-values are highlighted in italics

**Table 2:** Comparison of surgical outcomes at 6–12 months’ follow-up for short and traditional slings

	Type of sling surgery		
	Traditional slings^b^	Ajust™	TVT-A
	Reference	aOR (95% CI)	aOR (95% CI)
Primary outcomes^a^			
Objective failure	1.00	*2.86 (2.03–4.03)*	*1.42 (1.18–1.71)*
Subjective failure	1.00	*1.49 (1.17–1.91)*	*1.45 (1.28–1.65)*
Secondary outcomes^c^			
Treatment dissatisfaction	1.00	*1.73 (1.31–2.28)*	*1.32 (1.14–1.52)*
De novo urgency incontinence	1.00	*1.78 (1.01–3.12)*	1.10 (0.82–1.48)
Persistent urgency incontinence	1.00	1.18 (0.96–1.44)	1.11 (0.99–1.23)
Worsening urgency incontinence	1.00	*1.55 (1.13–2.11)*	*1.21 (1.01–1.44)*
Complications (total)	1.00	*0.37 (0.22–0.62)*	*0.75 (0.62–0.91)*
Vaginal erosions	1.00	0.47 (0.15–1.49)	1.05 (0.71–1.54)
Prolonged pain > 3 months	1.00	1.74 (0.70–4.34)	*1.96 (1.27–3.01)*

*aOR* adjusted odds ratios, *BMI* body mass index, *CI* confidence intervals, *TVT-A* tension-free vaginal tape obturator Abbrevo™

^a^Odds ratios for primary outcomes were adjusted for BMI at the time of surgery (WHO classification), age at time of surgery (years), hospital annual surgical volume, preoperative urgency incontinence symptom load, preoperative stress incontinence symptom load, preoperative objective leakage and total complication rate of complications occurring at or immediately after surgery (definitions given in the Materials and Methods section)

^b^Traditional slings (retropubic TVT and TVT-O)

^c^The secondary outcome of de novo urgency incontinence, persistent urgency incontinence and worsening urgency incontinence was adjusted for all of the above except preoperative urgency incontinence symptom load; the secondary outcomes complications, vaginal erosions and prolonged pain were adjusted for all the above except for complications (total)

Significant *p*-values are highlighted in italics

**Table 3:** Surgical and post-surgical complications reported prospectively to the Norwegian Female Incontinence Registry

Complication	Total population, *N* = 21,995 (%)	Traditional slings^f^, *N* = 18,612 (%)	TVT-A, *N* = 2,772 (%)	*p* value	Traditional slings ^f^, *N* = 18,612 (%)	Ajust™, *N* = 611 (%)	*p* value*
Bladder perforation	311 (1.4)	309 (1.7)	2 (0.1)	* < 0.01*	309 (1.7)	0 (0)	* < 0.01*
Deep infection ^a^	73 (0.3)	70 (0.4)	2 (0.1)	*0.01*	70 (0.4)	1 (0.2)	0.73
Superficial infection ^b^	137 (0.6)	132 (0.7)	3 (0.1)	* < 0.01*	132 (0.7)	2 (0.3)	0.45
Vaginal erosion	349 (1.6)	293 (1.6)	53 (1.9)	0.19	293 (1.6)	3 (0.5)	*0.03*
Prolonged pain ^c^	178 (0.8)	135 (0.7)	38 (1.4)	* < 0.01*	135 (0.7)	5 (0.8)	0.81
Hematoma^d^	167 (0.8)	161 (0.9)	5 (0.2)	* < 0.01*	161 (0.9)	1 (0.2)	0.06
Urinary retention:							
Sling “pull-down”	479 (2.4)	437 (2.6)	38 (1.4)	* < 0.01*	437 (2.6)	4 (0.7)	* < 0.01*
Catheterization > 1 week	367 (1.7)	310 (1.7)	44 (1.6)	0.76	310 (1.7)	13 (2.1)	0.38
Catheterization > 1 month	172 (0.8)	154 (0.8)	15 (0.5)	0.11	154 (0.8)	3 (0.5)	0.49
Sling transection	167 (0.8)	154 (0.9)	11 (0.4)	*0.02*	154 (0.9)	2 (0.3)	0.16
Other ^e^	65 (0.3)	57 (0.3)	5 (0.2)	0.25	57 (0.3)	3 (0.5)	0.44
Total	1,939 (8.8)	1,728 (9.3)	181 (6.5)	*<0.01*	1,728 (9.3)	30 (4.9)	* < 0.01*

*TVT-A* tension-free vaginal tape obturator Abbrevo™

*Chi-squared test and Fisher’s exact test when appropriate

^a^Abscess formation with or without sinus tract formation/Clavien–Dindo grade 3

^b^Local tenderness with tenderness and/or purulent discharge/Clavien–Dindo grade 2

^c^Prolonged pain defined as > 3 months post-surgery

^d^Clinically relevant hematoma defined by the Norwegian Female Incontinence Registry as > 4 cm

^e^Other rare complications grouped together: major vessel injury, major bleeding (> 500 ml), urethral injury and bowel injury/Clavien–Dindo grades 3 and 4

^f^Traditional sling (retropubic tension-free vaginal tape and tension-free vaginal tape obturator

Significant *p*-values are highlighted in italics

Traditional slings had a significantly higher incidence of bladder perforation and urinary retention solved by sling pull-down than both types of shorter slings (*p* < 0.01, Table 3). Traditional slings also had a higher incidence of sling transection than the shorter slings, but the number of women needing sling transection was small in all groups. There were no differences between sling types regarding urinary retention solved by intermittent catheterization. Traditional slings had a higher incidences of deep and superficial infection and hematoma than TVT-A but not Ajust™ (Table 3). Vaginal erosions were more often registered after traditional slings than after Ajust™ application, but not after TVT-A (Table 3). No group differences were seen of any severity of persistent urgency incontinence among women with pre-existing mixed incontinence: Ajust™ 38.2%, TVT-A 36.2%, and traditional slings 36.8% (*p* = 0.64). However, the rate of de novo urgency was significantly higher in the Ajust™ group: 24.1% vs 14.2% (TVT-A) vs 14.7% (traditional slings; (*p* < 0.01). The same was true for worsening of pre-existing urgency incontinence symptoms: 15.0% (Ajust™), 11.4% (TVT-A), and 10.3% (traditional slings; (*p* < 0.01).

The adjusted ORs for the outcomes treatment dissatisfaction, complications (total), vaginal erosions, prolonged postoperative pain, de novo urgency incontinence, persistent urgency incontinence, and worsening of urgency incontinence are given in Table 2. When analyzing complications separately for TVT and TVT-O versus shorter slings, the significant differences found were mostly ascribed to TVT (see Supplementary Tables 2A and B).

## Discussion

This large registry study demonstrates good short-term continence outcomes for all evaluated sling types. However, the shorter slings Ajust™ and TVT-A was found to have higher failure rates 6–12 months after surgery than traditional slings. To our knowledge, only the shorter sling, TVT-Secure, has previously been shown to have inferior cure rates to traditional slings [24, 25]. TVT-Secure is no longer on the market because of this lack of effectivity. The shorter slings Ajust™ and TVT-A have on the contrary in several studies, such as Zullo et al. and Abdel-Fattah et al., shown equally good short-term cure rates compared with traditional slings [19, 26]. The difference between our findings and these randomized controlled trials (RCTs) may be related to sample size and study design. RCTs entail strict inclusion and exclusion criteria whereas a registry study by design implies more heterogeneity in patient characteristics, department sizes, and surgeons with different levels of training. Therefore, even though the RCT by Abdel-Fattah and co-workers was a multicenter study including 21 hospitals, a large retrospective study adds knowledge by its amount of data and patient diversity, which may arguably be closer to a real-life clinical situation [19].

In our study women operated on with shorter slings were significantly more often less than “very satisfied” with their treatment, answering “moderately satisfied” or worse (17.5–18.3% vs 14.3, *p* < 0.01). This is comparable with what has been shown in a large multicenter RCT by Rudnicki and co-workers, in which 72.7% Ajust™-operated women and 82.2% traditional sling-operated women answered that they were “very satisfied” with treatment [13]. However, our study showed significantly higher subjective satisfaction with the treatment than a similar study by Canel and co-workers (TVT-O 66% vs TVT-A 68%) [27]. This might be ascribed to differences in the validated questionnaires involved, as we used the validated questionnaire developed by the NFIR, whereas Canel et al. used the Patient Global Impression of Improvement (PGI-I) questionnaire [22, 27].

Much focus has been placed on the risk of prolonged pain after synthetic sling surgery [9], and prior studies have shown that shorter slings with less synthetic material may cause less postoperative pain [13, 27]. However, the present study demonstrates that even though the incidence of prolonged postoperative pain was low in all groups, this serious complication occurred just as often after shorter slings as after traditional slings. This implies that mechanisms other than the length of the sling are important for causing prolonged pain, such as proximity to nerves, mechanical rubbing against the periosteum and soft-tissue irritation. In our study, the highest incidence of prolonged postoperative pain was found among women after TVT-A (1.4%). This contrasts some other studies showing reduced postoperative pain with shorter slings [13, 27]. A possible explanation might be that because TVT-A is a modification of the TVT-O and both sling types are placed using a transobturator technique [14], the risk of prolonged pain is related to the obturator route and not the length of the tape. It is, however, surprising that the Ajust™ performed better than TVT-A concerning prolonged pain, as the Ajust™ is also placed in the obturator region and is anchored in the obturator membrane. One may suspect that pain is prevented because the Ajust™ sling is not passed further into the pelvis beyond the obturator membrane. The nerve proximity in the obturator foramen could explain why in several systematic reviews the TVT-O has been shown to cause more postoperative pain than retropubic slings [1, 8]. Even though we included TVT-O in the traditional sling group, this group was dominated in number by retropubic TVTs, which might have diluted the pain impact from obturator slings.

One might speculate that women with low BMI are more prone to prolonged pain after TVT-A because less fat protects the surrounding tissues and more synthetic sling material might be deposited lateral to the obturator membrane in much the same way as a traditional transobturator sling. In a publication by Cadish and co-workers in 2010 it was shown that high BMI lowered the risk of prolonged postoperative pain after transobturator sling application [28]. However, the same research group later found the opposite association when studying a different cohort of patients, in which women with high BMI more often had prolonged pain after transobturator slings [29]. From subgroup analyses of women with BMI < 25 kg/m^2^ and < 20 kg/m^2^ in our data, we were not able to reveal any increased incidence of prolonged pain among slim women for either sling type. Apart from prolonged postoperative pain, our study demonstrated fewer overall complications with shorter slings than with traditional slings, which is in line with other studies [13, 26]. More bladder perforations, and more urinary retention solved by sling pull-down and transection, were as expected in the traditional slings as a part of this group consists of TVT slings, and these are known complications of the retropubic route [1, 8]. We also found an increased rate of vaginal erosions after TVT-A application compared to TVT-O, and this is surprising as other study groups have not been able to demonstrate any difference in erosion rates comparing TVT-A with traditional slings  [26].

Our study showed higher levels of de novo urgency incontinence for all slings than several other studies [13, 18, 19]. As we wanted to show all potential impacts on bladder symptoms, we chose a very strict definition for de novo urgency in which any, not just bothersome, symptoms of urgency urinary incontinence were considered relevant. An urgency incontinence index of 1–2 according to the index generated by the NFIR questionnaire is rarely regarded as bothersome by the patient; thus, including them probably explains the high incidence of urgency. Other studies showing a lower incidence of de novo urgency incontinence have used other definitions of bothersome incontinence [13, 18, 19]. However, the choice of definition does not explain the increased incidence among Ajust™ compared with the other slings, and we have no plausible explanation for this.

A registry study does have inherent limitations, such as potentially incorrect entries and missing data for certain variables. However, the NFIR was shown to have high accuracy, completeness, and coverage in a previous study by Dyrkorn and co-workers [20]. No imputation statistics was performed for missing values. The missing values were similarly distributed among groups, and therefore the likelihood of missing values introducing selection bias was considered low. Another limitation is possible selection bias caused by the NFIR only releasing data on women who had consented to their data being used for scientific purposes. According to the NFIR administration, the overall rate of women not consenting is 15%. However, the rate is not evenly distributed among reporting hospitals, and therefore we cannot exclude this having an impact on the results. Furthermore, the registry has until recently only recorded re-operations at the same hospital department. This means that data on previous incontinence surgeries at other hospitals are not available. However, there are few women with previous incontinence surgeries and they would be evenly distributed among groups. For the above reason, it was not possible to compare the rate of new incontinence surgery as a measure of treatment failure.

The NFIR questionnaire only includes one question concerning pain, and because pain is a subjective perception, this may have limited the quality of data on prolonged postoperative pain. The registry only records prolonged postoperative pain as lasting more than 3 months and not whether this has been resolved later.

The most important strength of this study is the large number of women included, which gives power to the statistical analyses and reduces risks of selection bias. As our study is based on national registry data, the external validity is high because it contains data from multiple surgeons with different levels of surgical training and all types of patients. The use of a validated questionnaire further strengthens the validity of the findings.

## Conclusion

The present study demonstrates good subjective and objective continence outcomes for all investigated slings, with low 6– to 12-month failure rates. Even though the shorter slings had significantly higher failure rates, the differences were small and probably of minimal clinical significance. In contrast to the slightly inferior effectiveness of the shorter slings, they do seem to cause fewer overall complications. Still the Ajust™ showed higher rates of de novo urgency incontinence and the TVT-A higher rates of prolonged pain. When analyzing complications separately for the two traditional slings, TVT had the highest overall complication rate and the TVT-O was more comparable with the shorter slings. The optimal type of surgery has therefore still not been found, and the decision whether to use a traditional sling or a shorter sling should be made after careful deliberation between an informed patient and her doctor regarding effect and complication risks. Our study shows that the incidence of prolonged postoperative pain is low for all slings, something we believe must be communicated to health authorities because the use of synthetic slings for stress urinary incontinence is under threat of being withdrawn. Furthermore, well-designed studies to investigate the long-term efficacies and rates of prolonged postoperative pain of various slings are urgently needed.

## Electronic supplementary material

Below is the link to the electronic supplementary material.

Supplementary file1 (DOCX 15 KB)

Supplementary file2 (DOCX 17 KB)

Supplementary file3 (DOCX 17 KB)

## Data Availability

All our main findings is shown in figures and tables; Figure 1 showing a flow chart of the exclusion criteria of FAST 1, Table 1 with baseline characteristics of study participants at the time of surgery, Table 2 with comparison of surgical outcomes at 6-12 months` follow-up for short and traditional slings, Table 3 with surgical and post-surgical complications reported prospectively to the NFIR, Supplementary Table 1 with subgroup analysis of low BMI, sling-type and prolonged postoperative pain, Supplemantary Table 2a with surgical and post-surgical complications reported prospectively to the Norwegian Female Incontinence Registry comparing TVT to the shorter slings TVT-A and Ajust^TM^, Supplementary Table 2b with surgical and post-surgical complications reported prospectively to the Norwegian Female Incontinence Registry comparing TVT-O to the shorter slings TVT-A and Ajust^TM^ as well as the full NFIR Questionnaire are all available to any reader wanting to know more about the findings in our study.
