# Supplementary material for: Effectiveness and Safety of Shorter Incontinence Slings
Source: Int Urogynecol J. 2024 Nov 13;36(1):135–45. doi: 10.1007/s00192-024-05971-5 (PMC11785704; doi:10.1007/s00192-024-05971-5)
Supplement: Supplementary file 1 — Supplementary file1 (DOCX 15 KB) [file 192_2024_5971_MOESM1_ESM.docx]

**Supplementary Table 1:** Subgroup analysis of low BMI, sling-type and prolonged postoperative pain

| **Type of sling-surgery** | **Body Mass Index (kg/m^2^)** | | | | | |
| --- | --- | --- | --- | --- | --- | --- |
|  | < 25.0 | ≥ 25.0 |  | < 20.0 | ≥ 20.0 |  |
|  | Prolonged postoperative pain  % (n/N) | | P-value^a^ | Prolonged postoperative pain  % (n/N) | | P-value^a^ |
| **Traditional slings^b^** | 0.7  (50/7344) | 0.8  (73/9726) | 0.59 | 0.9  (5/549) | 0.7  (118/16521) | 0.60 |
| **AJUST™** | 1.0  (2/197) | 1.1  (3/284) | 1.00 | 4.8  (1/21) | 0.9  (4/460) | 0.20 |
| **TVT-A^c^** | 0.9  (8/909) | 1.9  (25/1344) | 0.06 | 1.9  (1/52) | 1.5  (32/2201) | 0.54 |

^a^ Chi-square-test or Fisher’s Exact Test when appropriate

^b^Traditional slings (Retropubic Tension-free Vaginal Tape (TVT) and Tension-Free Vaginal Tape Obturator (TVT-O))

^c^TVT-A (TVT-O Abbrevo^TM^)
