# Supplementary material for: Effectiveness and Safety of Shorter Incontinence Slings
Source: Int Urogynecol J. 2024 Nov 13;36(1):135–45. doi: 10.1007/s00192-024-05971-5 (PMC11785704; doi:10.1007/s00192-024-05971-5)
Supplement: Supplementary file 2 — Supplementary file2 (DOCX 17 KB) [file 192_2024_5971_MOESM2_ESM.docx]

**Supplementary table 2A:** Surgical and post-surgical complications reported prospectively to the Norwegian Female Incontinence Registry

| **Complication** | **TVT**  **N = 16 769 (%)** | **TVT-A^a^**  **N = 2 772 (%)** | **P value^b^** | **TVT**  **N = 16 769 (%)** | **AJUST™**  **N = 611 (%)** | **P** **value ^b^** |
| --- | --- | --- | --- | --- | --- | --- |
| Bladder perforation | 302 (1.8) | 2 (0.1) | **< 0.01** | 302 (1.8) | 0 (0) | **< 0.01** |
| Deep infection ^c^ | 70 (0.4) | 2 (0.1) | **<0.01** | 70 (0.4) | 1 (0.2) | 0.52 |
| Superficial infection ^d^ | 129 (0.8) | 3 (0.1) | **< 0.01** | 129 (0.8) | 2 (0.3) | 0.34 |
| Vaginal erosion | 275 (1.6) | 53 (1.9) | 0.30 | 275 (1.6) | 3 (0.5) | **0.03** |
| Prolonged pain ^e^ | 118 (0.7) | 38 (1.4) | **< 0.01** | 118 (0.7) | 5 (0.8) | 0.62 |
| Hematoma^f^ | 158 (0.9) | 5 (0.2) | **< 0.01** | 158 (0.9) | 1 (0.2) | 0.05 |
| Urinary retention:  Sling “pull-down”  Catheterization > 1 week  Catheterization > 1 month  Sling transection | 412 (2.7)  288 (1.7)  148 (0.9)  141 (0.9) | 38 (1.4)  44 (1.6)  15 (0.5)  11 (0.4) | **< 0.01**  0.62  0.07  **0.02** | 412 (2.7)  288 (1.7)  148 (0.9)  141 (0.9) | 4 (0.7)  13 (2.1)  3 (0.5)  2 (0.3) | **< 0.01**  0.45  0.31  0.16 |
| Other ^g^ | 56 (0.3) | 5 (0.2) | 0.18 | 56 (0.3) | 3 (0.5) | 0.46 |
| **Total** | **1627 (9.7)** | **181 (6.5)** | **< 0.01** | **1627 (9.7)** | **30 (4.9)** | **< 0.01** |

^a^ TVT-A (TVT-O Abbrevo^TM^)

^b^ Chi-square-test and Fisher’s Exact Test when appropriate

^c^ Abscess formation with or without sinus tract formation/Clavien-Dindo grade 3

^d^ Local tenderness with tenderness and/or purulent discharge/Clavien-Dindo grade 2

^e^ Prolonged pain defined as > 3 months post-surgery

^f^ Clinical relevant hematoma defined by NFIR as > 4cm

^g^ Other rare complications here grouped together: major vessel injury, major bleeding (> 500 ml), urethral injury and bowel injury/ Clavien-Dindo grade 3 and 4
