# Supplementary material for: Effectiveness and Safety of Shorter Incontinence Slings
Source: Int Urogynecol J. 2024 Nov 13;36(1):135–45. doi: 10.1007/s00192-024-05971-5 (PMC11785704; doi:10.1007/s00192-024-05971-5)
Supplement: Supplementary file 3 — Supplementary file3 (DOCX 17 KB) [file 192_2024_5971_MOESM3_ESM.docx]

**Supplementary table 2B:** Surgical and post-surgical complications reported prospectively to the Norwegian Female Incontinence Registry

| **Complication** | **TVT-O**  **N = 1 843 (%)** | **TVT-A^a^**  **N = 2 772 (%)** | **P value^b^** | **TVT-O**  **N = 1 843 (%)** | **AJUST™**  **N = 611 (%)** | **P value ^b^** |
| --- | --- | --- | --- | --- | --- | --- |
| Bladder perforation | 7 (0.4) | 2 (0.1) | **0.04** | 7 (0.4) | 0 (0) | 0.20 |
| Deep infection ^c^ | 0 (0.0) | 2 (0.1) | 0.52 | 0 (0.0) | 1 (0.2) | 0.25 |
| Superficial infection ^d^ | 3 (0.2) | 3 (0.1) | 0.69 | 3 (0.2) | 2 (0.3) | 0.60 |
| Vaginal erosion | 18 (1.0) | 53 (1.9) | **0.01** | 18 (1.0) | 3 (0.5) | 0.26 |
| Prolonged pain ^e^ | 17 (0.9) | 38 (1.4) | 0.17 | 17 (0.9) | 5 (0.8) | 0.81 |
| Hematoma^f^ | 3 (0.2) | 5 (0.2) | 1.00 | 3 (0.2) | 1 (0.2) | 1.0 |
| Urinary retention:  Sling “pull-down”  Catheterization > 1 week  Catheterization > 1 month  Sling transection | 25 (1.6)  22 (1.2)  6 (0.3)  13 (0.8) | 38 (1.4)  44 (1.6)  15 (0.5)  11 (0.4) | 0.56  0.27  0.29  0.13 | 25 (1.6)  22 (1.2)  6 (0.3)  13 (0.8) | 4 (0.7)  13 (2.1)  3 (0.5)  2 (0.3) | 0.15  0.09  0.70  0.38 |
| Other ^g^ | 1 (0.1) | 5 (0.2) | 0.41 | 1 (0.1) | 3 (0.5) | 0.05 |
| **Total** | **101 (5.5)** | **181 (6.5)** | **0.15** | **101 (5.5)** | **30 (4.9)** | **0.59** |

^a^ TVT-A (TVT-O Abbrevo^TM^)

^b^ Chi-square-test and Fisher’s Exact Test when appropriate

^c^ Abscess formation with or without sinus tract formation/Clavien-Dindo grade 3

^d^ Local tenderness with tenderness and/or purulent discharge/Clavien-Dindo grade 2

^e^ Prolonged pain defined as > 3 months post-surgery

^f^ Clinical relevant hematoma defined by NFIR as > 4cm

^g^ Other rare complications here grouped together: major vessel injury, major bleeding (> 500 ml), urethral injury and bowel injury/ Clavien-Dindo grade 3 and 4
